# Middle cerebral artery bifurcation aneurysms are associated with patient age, sex, bifurcation angle, and vascular diameters

**DOI:** 10.1038/s41598-023-50380-1

**Published:** 2023-12-21

**Authors:** Shu Wang, Li Li, Huili Gao, Kun Zhang, Qiu-Ji Shao, Tianxiao Li, Bulang Gao

**Affiliations:** Stroke Center, Henan Provincial People’s Hospital, 7 Weiwu Road, Zhengzhou, 450000 Henan Province China

**Keywords:** Diseases, Health care, Medical research, Neurology

## Abstract

To investigate the relationship of the middle cerebral artery (MCA) bifurcation aneurysms with patients’ age and sex, vascular angles at the bifurcation, and diameters of the M1 and two M2 arteries, patients with and without MCA aneurysms were retrospectively enrolled. The lateral angles, MCA bifurcation angle and arterial diameter were measured and analyzed. Totally, 121 (19.0%) patients with and 517 (81.0%) without MCA aneurysms were enrolled. Most (n = 88 or 72.7%) aneurysms were present in the age range of 40–70 years, and significantly (*P* = 0.01) more women than men had the bifurcation aneurysms. The MCA bifurcation angle was significantly greater (149.2° ± 32.6° vs. 107.2° ± 26.3°; *P* < 0.0001) while both the smaller and larger lateral (M1/M2) angles were significantly smaller in patients with than without aneurysms (82.0° ± 23.7° vs. 109.1° ± 22.7° with *P* < 0.001 for the smaller and 123.2° ± 25.2° vs. 139.5° ± 16.9° with *P* < 0.001 for the larger lateral angle). 109 (90.1%) bifurcation aneurysms deviated towards the smaller lateral angle, and 103 (85.1%) aneurysms deviated towards the thinner M2 branch. The maximal aneurysm diameter ranged 1.6–13.8 (mean 5.4 ± 2.4) mm and was significantly (*P* < 0.05) positively correlated with the diameter of both M2 arterial branches (R = 0.57 and *P* = 0.01 for the smaller M2, and R = 0.69 and *P* = 0.002 for the larger M2) or the MCA bifurcation angle. A significant (*P* < 0.0001) negative correlation was detected between age and the smaller lateral angle but a significant (*P* < 0.0001) positive correlation between age and the MCA bifurcation angle in patients without MCA bifurcation aneurysms or in the total patients. MCA bifurcation angle was the only significant (*P* = 0.0001, odds ratio 2.7, 95% confidence interval 1.6–3.8) independent risk factor for MCA bifurcation aneurysm presence, with the bifurcation angle threshold of 124.1° and an area under the ROC curve of 0.86. In conclusion, significantly more MCA bifurcation aneurysms are present in older patients, females, and patients with a wider MCA bifurcation angle, and deviate towards the smaller lateral angle and the thinner M2 segment. MCA bifurcation angle is the only independent risk factor for presence of MCA bifurcation aneurysms with the threshold of 124.1°.

## Introduction

Intracranial aneurysms may affect 2–4% of the total population^1,2^, and the middle cerebral artery (MCA) bifurcation is a preferred location of aneurysm presence, accounting for 18–36% of all intracranial aneurysms^3^. It has a yearly rupture rate of 0.36%^4^. The MCA bifurcation aneurysms have complex branching arteries and are hard to be treated^5^. Clear understanding of the relationship of the MCA bifurcation aneurysms with patient age, sex and bifurcation morphology will help risk evaluation of aneurysm presence and treatment outcome because these factors have been revealed to affect the hemodynamic stresses responsible for aneurysm initiation^6–15^. The intracranial aneurysms at both bifurcations of the anterior cerebral artery and basilar artery are associated with old age, wider bifurcation angles, and smaller vascular diameter at the bifurcation^6,7^. Wider bifurcation angles of the anterior cerebral artery and basilar artery are associated with aneurysm presence probably through enhancing the hemodynamic stresses^6,7,15^. Aneurysms at these arterial bifurcations deviated mostly towards the smaller lateral angle and thinner arterial branch^6,7^. Hemodynamic stresses play an important role in the distribution of work at cerebral arterial bifurcations, and presence of bifurcation aneurysms may be predicted by arterial branch angles^9^. Normal anterior cerebral artery bifurcations obey the optimality principle whereas aneurysmal bifurcations do not obey the optimality principle and present significantly enhanced hemodynamic stresses to damage the bifurcation wall for aneurysm initiation^10^. Moreover, enlarged anterior cerebral artery bifurcation angles have been demonstrated to be able to induce abnormally enhanced hemodynamic stresses to initiate aneurysms^11^, and greater hemodynamic stresses are able to initiate an anterior communicating artery aneurysm on the vascular bifurcation apex^15^. Clinical application of Y-stent coiling and single stent-assisted coiling can significantly decrease the arterial bifurcation angle and, thus, significantly lower the bifurcation hemodynamic stresses which may protect the bifurcation apex from being damaged by high stresses, resulting in endured treatment effect^8,14^. MCA bifurcations with aneurysms have been shown to have significantly larger branching angles and more often originate off the branch with the largest angle, which may indicate altered wall shear stress regulation as a possible factor in aneurysm development and progression^13^. Moreover, it has been revealed that hemodynamic stresses may induce initially endothelial dysfunction followed by inflammatory reactions in arterial walls, involving mainly macrophages and smooth muscle cells, and finally a degradation of the extracellular matrix, which paves the way for aneurysm initiation, enlargement and rupture^12^. Nonetheless, no sufficient studies have been conducted on the association of MCA bifurcation aneurysms with relevant clinical data of the patients, including age, sex, arterial diameters, lateral and bifurcating angles at the MCA, and direction of aneurysm deviation either towards a smaller lateral angle or a thinner M2 segment in a relatively large cohort of patients. It was hypothesized that the MCA bifurcation aneurysms were associated with patient age, sex, certain direction of aneurysm deviation, and bifurcation morphology, including arterial lateral and bifurcation angles and diameters. This study was consequently performed to test this hypothesis.

## Materials and methods

This one-center retrospective study was approved by the ethics committee of Henan Provincial People’s Hospital (ID 20,210,218), and informed consent was waived by the same ethics committee of Henan Provincial People’s Hospital because of the retrospective study design. All methods were conducted in accordance with the relevant guidelines and regulations. Between January 2017 and January 2023, consecutive patients with and without MCA bifurcation aneurysms were enrolled. The inclusion criteria were patients with MCA bifurcation aneurysms confirmed by cerebral angiography (computed tomographic, magnetic resonance imaging or digital subtraction angiography), sufficient clinical data for analysis, availability of three-dimensional cerebral angiography in the format of DICOM, and clear angiographic imaging for morphological measurement. The exclusion criteria were patients without MCA bifurcation aneurysms or with MCA bifurcation aneurysms who did not have sufficient relevant clinical data, three-dimensional cerebral angiography or clear angiographic imaging for morphological measurement and analysis. Patients without any cerebral aneurysms were enrolled as the control group. The MCA bifurcation aneurysms are intracranial aneurysms located at the M1 (MCA trunk) bifurcating apex where two M2 segments bifurcate, which was confirmed by computed tomographic angiography, magnetic resonance imaging or digital subtraction angiography.

**Figure 1:**
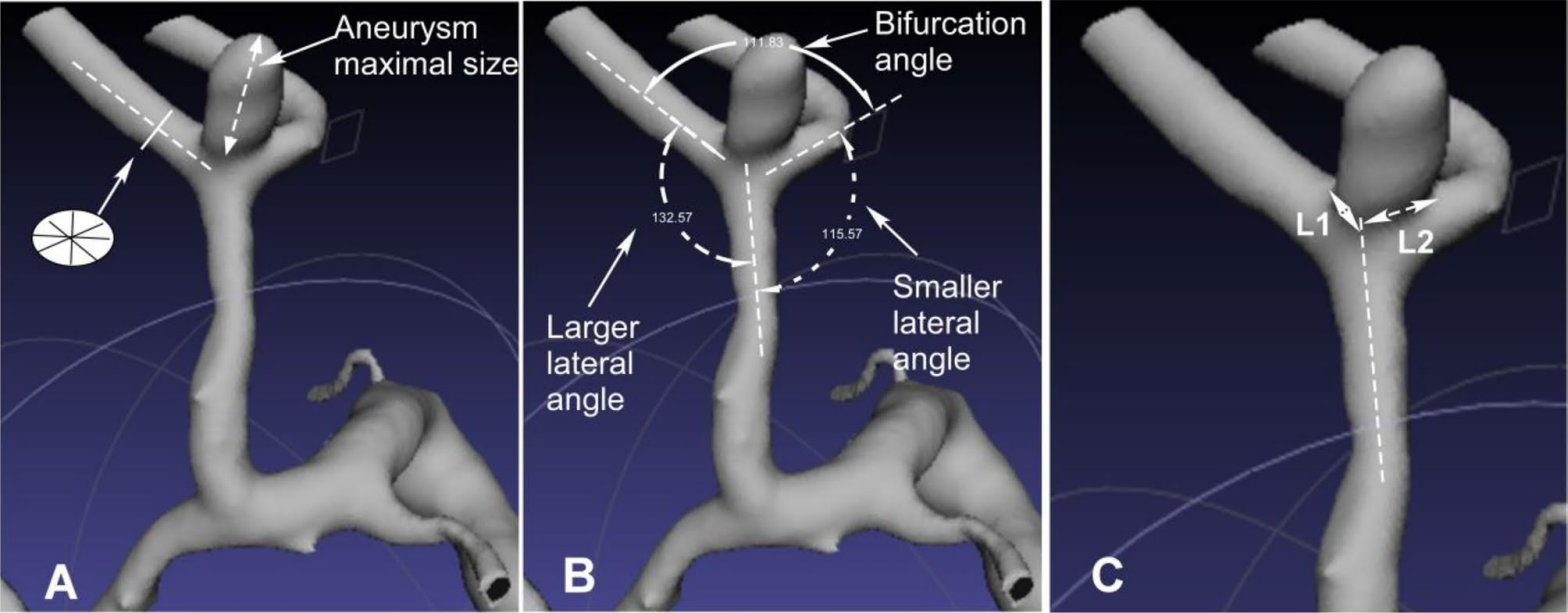
Measurement of vascular diameter, angles and aneurysm deviation at the middle cerebral artery (MCA) bifurcation. (**A**) The arterial diameter was measured 5 mm away from the MCA bifurcation apex four times for calculation of the average value. The maximal aneurysm size indicated the maximal length of the aneurysm dome. (**B**) The smaller and larger lateral angle and bifurcation angle were measured at the MCA bifurcation. (**C**) Aneurysm deviation was measured by dividing the aneurysm neck length into two sections by the M1 central axis: L1 and L2. If L1 was > L2, the aneurysm deviated towards the L1 side. Otherwise, it deviated towards the L2 side. In this case, the aneurysm deviated towards the L2 and smaller lateral angle side.

Measurement of the morphological data was performed using the Amira software (version 5.2.2, Visage Imaging, San Diego, CA, USA) for three-dimensional imaging reconstruction and measurement and the three-dimensional imaging data in the DICOM format. The DICOM data were imported into the Amira software. The diameter was measured on the MCA M1 (MCA trunk) and M2 segments 5 mm away from the MCA bifurcation. A cutting plane perpendicular to the central axis of the arterial segment was applied, and the diameter of the MCA artery at the M1 segment (the MCA trunk) and two M2 segments was measured on the cutting plane four times with the average value being calculated as the diameter value of each segment (Fig. 1A). The DA ratio indicated the ratio of the arterial diameter between the larger M2 and smaller M2 (larger/smaller M2 diameter) segment and was also measured. The aneurysmal maximal size indicated the maximal diameter of the aneurysm dome (Fig. 1A) and was measured in the three-dimensional Arimira software. The MCA bifurcation angles were measured (Fig. 1B) in the three-dimensional space. The MCA bifurcation angle indicated the angle formed between two M2 segments, and two lateral bifurcation angles were the angles formed by the M1 segment with each M2 segment (Fig. 1B). In measuring the angle formed by two M2 arterial branches or the M1 and one M2 segment, a central axis of each arterial segment or branch was established, and the angle formed between two central axes (standing for two segments) were measured using the Canvas software (GFX, Inc. Boston, MA, USA). The LA ratio indicated the ratio between the larger lateral angle and smaller lateral angle (larger/smaller lateral angle). Aneurysm deviation was measured according to the method stated by Zhang et al.^6,7^. The aneurysm neck was divided by the central axis of the M1 segment into two sections: L1 and L2. In aneurysms with L1 > L2, the aneurysm deviated towards the L1 side. Otherwise, the aneurysm deviated towards the L2 side (Fig. 1C). Two authors with experience of imaging analysis over 10 years measured the data independently and if in disagreement, a third senior was involved to reach a consensus.

### Statistical analysis:

The JMP software (Version 10.01, SAS, Cary, NC, USA) was used for the statistical analysis in this study. Measurement data in the normal distribution were presented in the form of mean ± standard deviation and tested with the t-test or in the form of median and interquartile range if not in the normal distribution and tested with the Mann Whitney U test. Categorical data were presented in frequency and percentage and tested with the Chi square test. Correlation was calculated between different parameters. Univariate and multivariate logistic regression analysis was conducted for the risk factors of MCA bifurcation aneurysms. Receiver operating characteristics (ROC) curve analysis was performed for significant continuous risk factors for the presence of MCA bifurcation aneurysm. The significant *P* was set at < 0.05.

## Results

**Figure 2:**
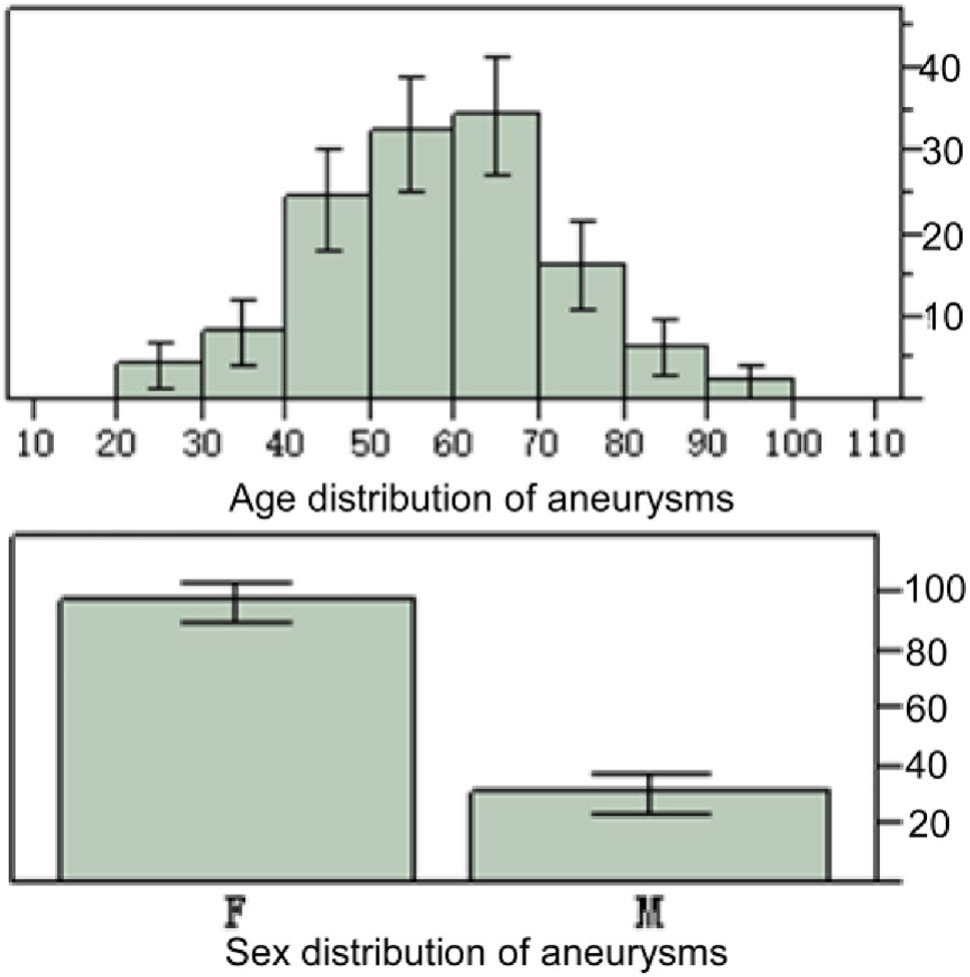
Age and sex distribution of the middle cerebral artery (MCA) bifurcation aneurysms. Most aneurysms were present in the age range of 40–70 years, and significantly (*P* = 0.01) more women than men had the bifurcation aneurysms.

A total of 638 patients with or without MCA bifurcation aneurysms were enrolled, including 121 (19.0%, F/M = 89/32) patients with MCA aneurysms aged 21- 92 (58.1 ± 13.7) years and 517 (81.0%, F/M = 351/155) without any cerebral aneurysms aged 18–88 (55.2 ± 13.8) years (Table 1). No significant (*P* > 0.05) differences were detected in the age and sex ratio between patients with and without aneurysms. However, most (n = 88 or 72.7%) aneurysms were present in the age range of 40–70 years, and significantly (*P* = 0.01) more women than men had the bifurcation aneurysms (Fig. 2).

**Table 1:** Data in patients with and without MCA bifurcation aneurysms.

Variables	Aneurysm patients (121)			Patients without aneurysms (517)			*P*
	Total	F	M	Total	F	M	
Age (y)	21–92 (58.1 ± 13.7)	57.40 ± 14.52	60.53 ± 10.44	18–88 (55.2 ± 13.8)	56.23 ± 13.97	54.99 ± 13.24	0.86
F/M	89/32	NA	NA	351/155	NA	NA	NA
MCA bifurcation angle (°)	51.4–247.8 (149.2 ± 32.6)	147.9 ± 31.7	153.5 ± 37.1	40–229 (107.2 ± 26.3)	106.78 ± 26.37	108.13 ± 26.90	< 0.0001
Smaller M1-M2 angle (°)	46.1–163.3 (82.0 ± 23.7)	85.0 ± 24.09	72.37 ± 20.08	13.7–173.8 (109.1 ± 22.7)	109.25 ± 23.47	109.99 ± 21.05	< 0.0001
Larger M1-M2 angle (°)	58.8–169.5 (123.2 ± 25.2)	124.45 ± 24.25	121.95 ± 28.19	71.8–177.2 (139.5 ± 16.9)	139.17 ± 16.96	139.38 ± 16.34	< 0.001
LA ratio	0.4–3.5 (1.7 ± 0.6)	1.6 ± 0.6	1.85 ± 0.8	0.5–8.4 (1.4 ± 0.5)	1.29 ± 0.51	1.26 ± 0.58	0.007
M1 diameter (mm)	1.6–5.1 (2.7 ± 0.9)	2.45 ± 0.89	2.89 ± 0.41	1.3–6.3 (3.2 ± 1.3)	3.11 ± 1.30	3.37 ± 1.39	0.03
Smaller M2 diameter (mm)	1.25–3.55 (2.2 ± 0.6)	2.12 ± 0.50	2.10 ± 0.65	0.6–5.4 (2.3 ± 0.9)	2.19 ± 0.83	2.39 ± 0.98	0.06
Larger M2 diameter (mm)	0.9–7.1 (2.6 ± 1.2)	2.27 ± 0.59	2.9 ± 0.98	0.7–8.2 (2.7 ± 1.2)	2.65 ± 1.21	2.97 ± 1.31	0.16
DA ratio	0.4–2.0 (1.2 ± 0.4)	1.1 ± 0.3	1.4 ± 0.3	0.3–4.5 (1.3 ± 0.5)	1.29 ± 0.51	1.26 ± 0.58	0.76
Aneurysm size (mm)	1.6–13.8 (5.4 ± 2.4)	5.39 ± 2.25	4.64 ± 1.95	NA	NA	NA	NA

No significant (*P* > 0.05) sex difference existed in the data between the females and males in the data in patients with or without aneurysms. P indicated the comparison between the total data of aneurysm patients and patients without aneurysms.

*MCA* Middle cerebral artery.

The MCA bifurcation angle was significantly greater in patients with than without aneurysms (149.2° ± 32.6° vs. 107.2° ± 26.3°; *P* < 0.0001), whereas both the smaller and larger lateral (M1/M2) angles were significantly smaller in patients with than those without aneurysms (82.0° ± 23.7° vs. 109.1° ± 22.7° with the *P* < 0.001 for the smaller and 123.2° ± 25.2° vs. 139.5° ± 16.9° with the *P* < 0.001 for the larger lateral angle). The LA ratio (larger/smaller lateral angle) was significantly (*P* = 0.007) greater in patients with than those without MCA bifurcation aneurysms (1.7 ± 0.6 vs. 1.4 ± 0.5). The MCA M1 diameter was significantly smaller in patients with than those without aneurysms (2.7 ± 0.9 vs. 3.21.3 mm; *P* = 0.03), but no significant differences were detected in the diameter of the two M2 segments at either the smaller (2.2 ± 0.6 vs. 2.3 ± 0.9 mm; *P* > 0.05) or larger (2.6 ± 1.2 vs. 2.7 ± 1.2 mm; *P* > 0.05) lateral angle side. The DA ratio was not significantly different between patients with and without aneurysms (1.2 ± 0.4 vs. 1.3 ± 0.5; *P* = 0.76).

**Figure 3:**
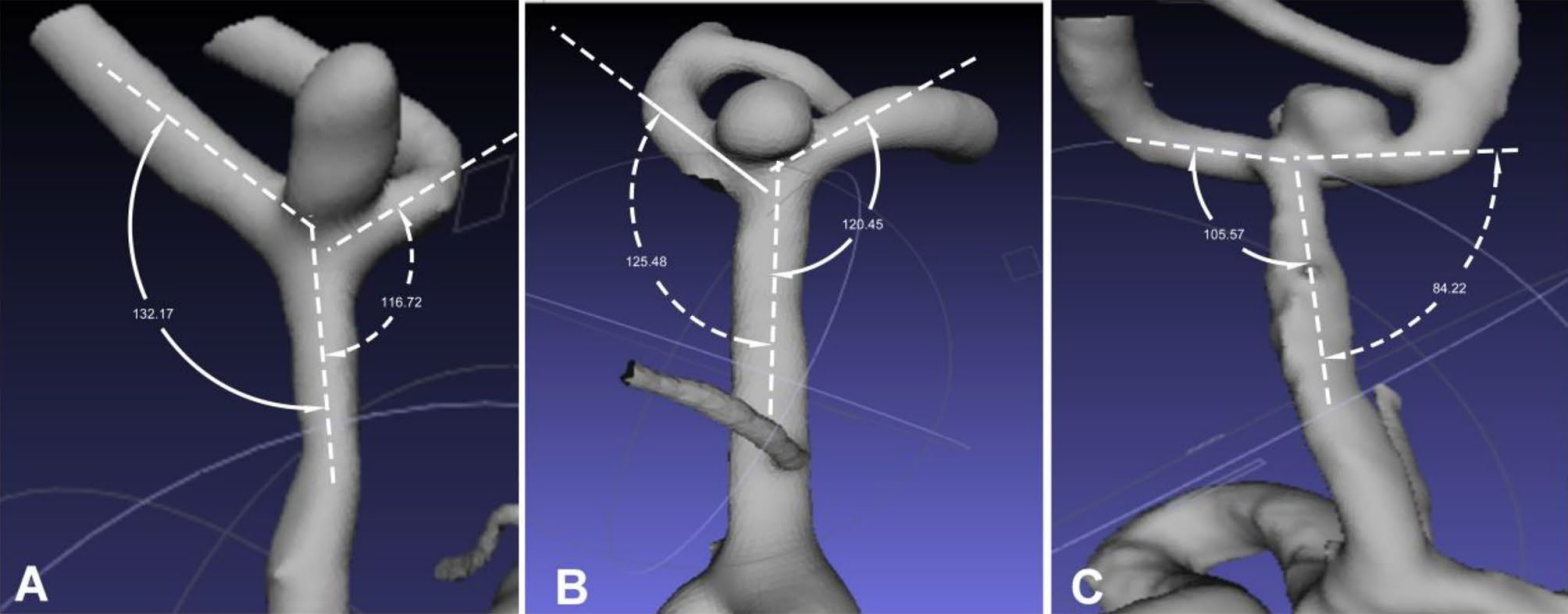
Aneurysm deviation towards different angles and arteries with different sizes. (**A**) The middle cerebral artery (MCA) bifurcation aneurysm deviated towards the smaller lateral angle and thinner M2. (**B**) The MCA bifurcation aneurysm deviated towards the larger lateral angle and larger M2. (**C**) The MCA bifurcation aneurysm deviated towards the smaller lateral angle but larger M2.

Among patients with MCA bifurcation aneurysms, 109 (90.1%) aneurysms deviated towards the smaller lateral angle, and 103 (85.1%) aneurysms deviated towards the thinner M2 branch (Fig. 3).

**Figure 4:**
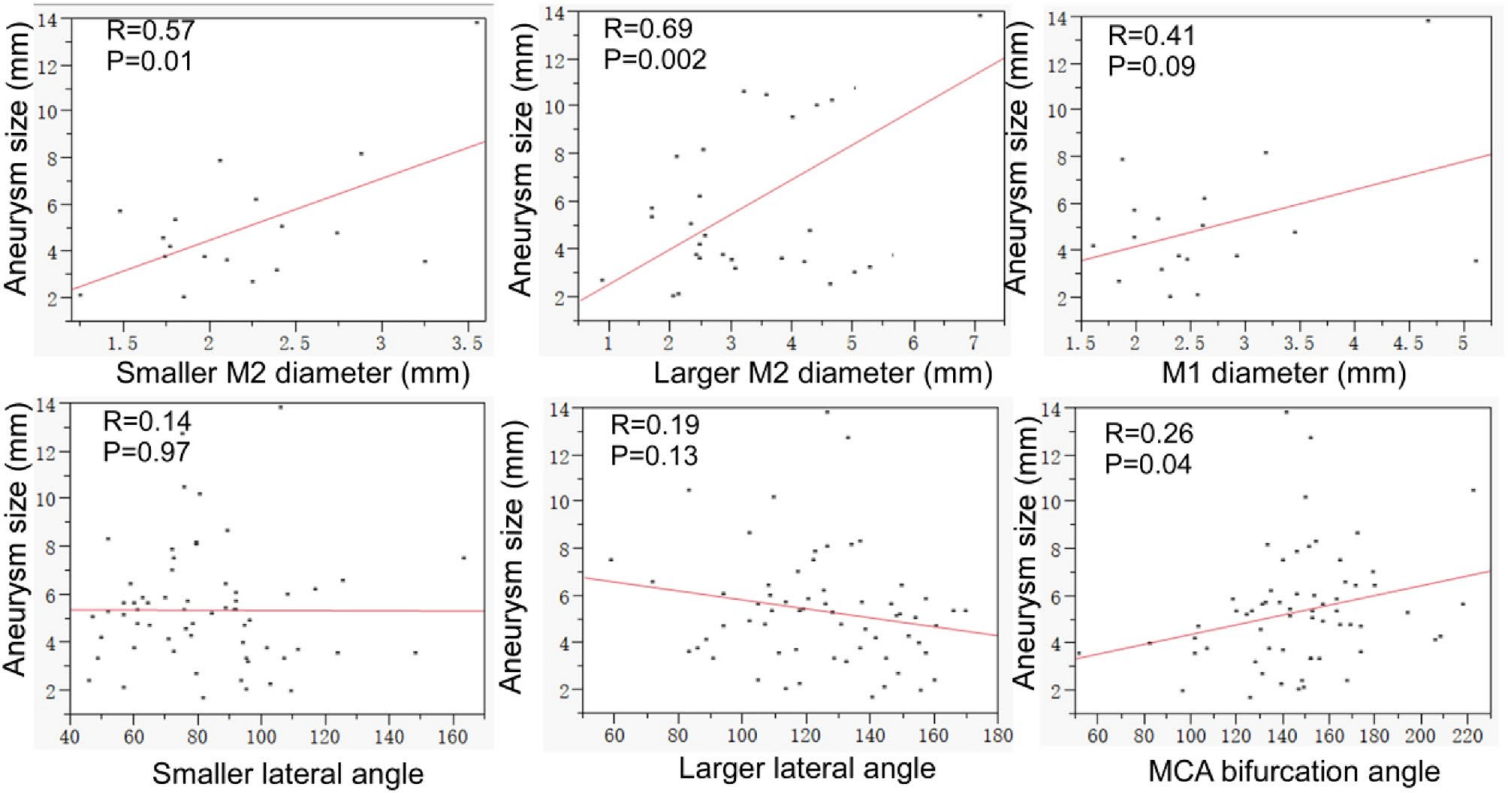
Correlation of the arterial diameters and angles with the maximal aneurysm size at the middle cerebral artery (MCA) bifurcation. The MCA bifurcation aneurysm maximal size was significantly (*P* = 0.01 or < 0.0001) positively correlated with the diameter of the smaller and larger M2 branches rather than the M1 artery. For the angles, the aneurysm maximal size was not significantly (*P* > 0.05) correlated with the smaller or larger lateral angles but was significantly (*P* = 0.04) positively correlated with the MCA bifurcation angle formed between two M2 branches.

The maximal aneurysm diameter ranged 1.6–13.8 (mean 5.4 ± 2.4) mm and was not significantly (*P* = 0.26) different between women and men (mean 5.4 vs. 4.6 mm). The maximal aneurysm size was significantly (*P* < 0.05) positively correlated with the diameter of both M2 arterial branches (R = 0.57 and *P* = 0.01 for the smaller M2, and R = 0.69 and *P* = 0.002 for the larger M2) but was not significantly (*P* = 0.09) correlated with the M1 diameter (Fig. 4). The aneurysm size was also significantly (*P* = 0.04) positively correlated with the MCA bifurcation angle rather than with both smaller and larger lateral angles (*P* > 0.05) (Fig. 4). The maximal aneurysm size was not significantly (*P* > 0.05) correlated with patient age (R = 0.22, *P* = 0.09).

**Figure 5:**
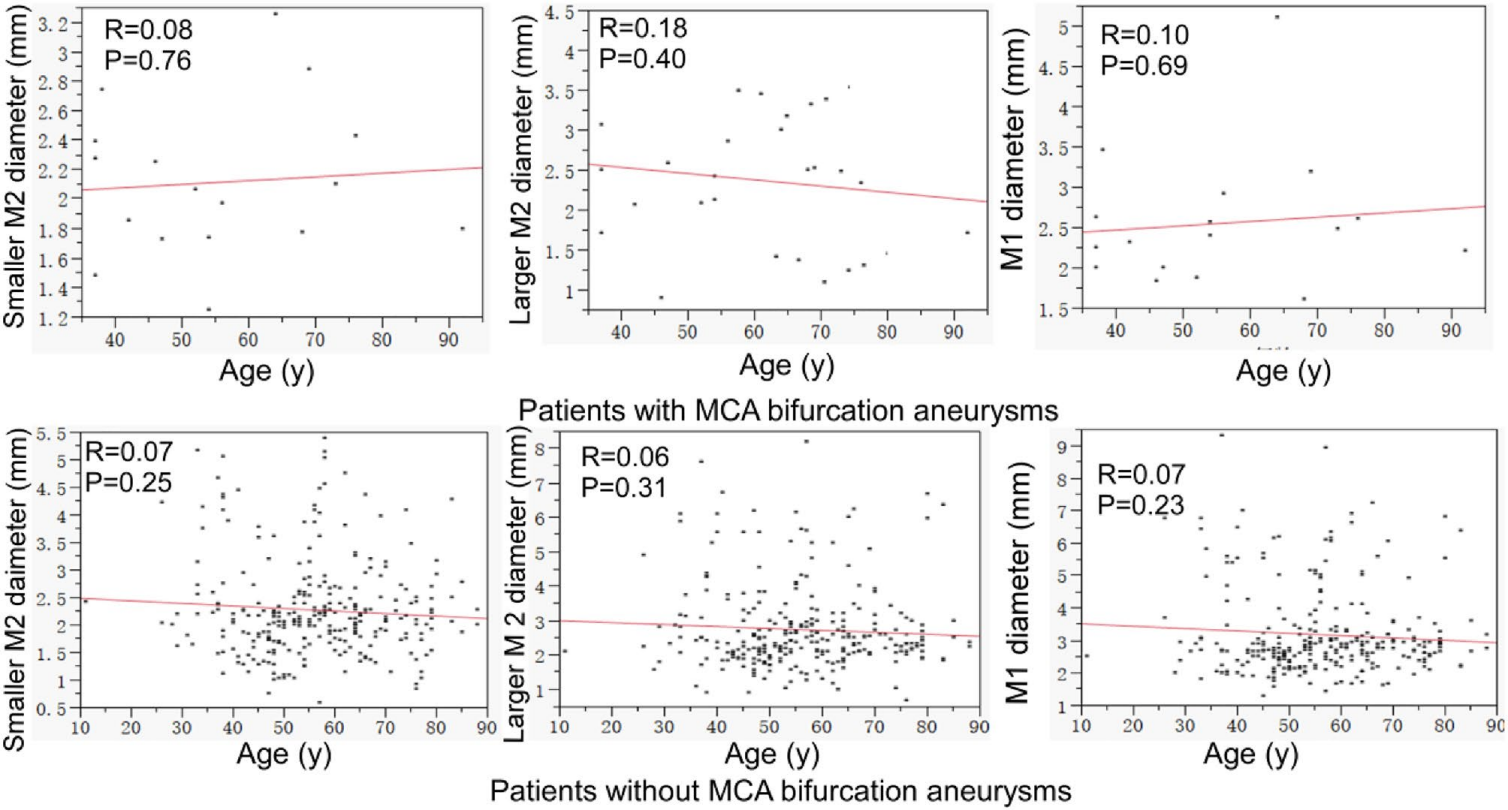
Correlation between age and the diameter of M1 and smaller and larger M2 in patients with or without middle cerebral artery (MCA) bifurcation aneurysms. No significant (*P* > 0.05) correlation was detected between the age (years) and the diameter of M1 and smaller and larger M2 with or without aneurysms.

In patients with MCA bifurcation aneurysms, age was not significantly (*P* > 0.05) correlated with the diameter of the smaller M2 (R = 0.08 and *P* = 0.76), larger M2 (R = 0.18 and *P* = 0.40) or M1 (R = 0.10 and *P* = 0.69) (Fig. 5). In patients without any aneurysms, age was not significantly (*P* > 0.05) correlated with the diameter of the smaller M2 (R = 0.07 and *P* = 0.25), larger M2 (R = 0.06 and *P* = 0.31), or M1 (R = 0.07 and *P* = 0.23).

**Figure 6:**
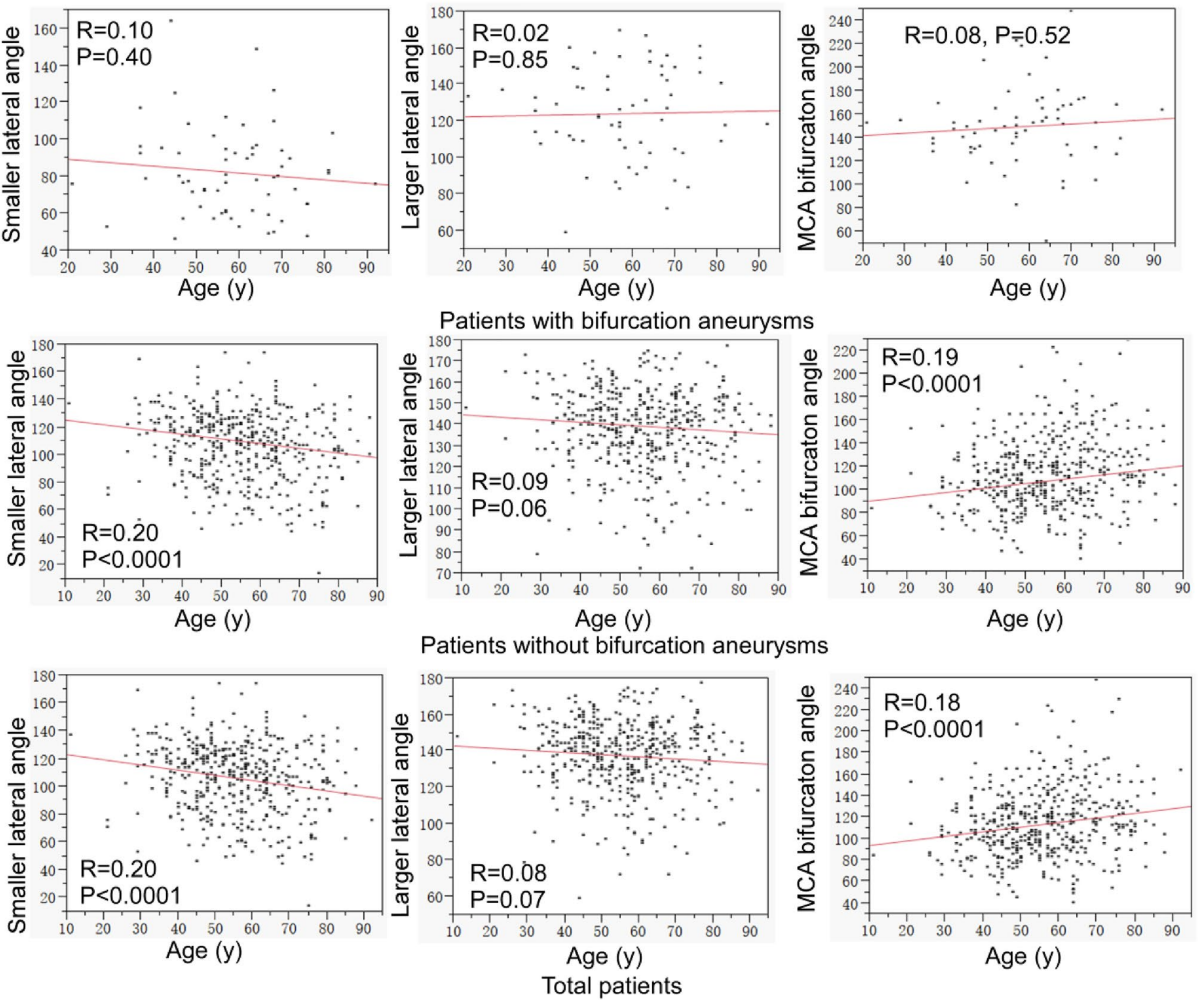
Correlation between age (years) and the middle cerebral artery (MCA) bifurcation angle or the smaller or larger lateral angle. No significant (*P* > 0.05) correlation was detected between age and the vascular angles in patients with MCA bifurcation aneurysms. A significant (*P* < 0.0001) negative correlation was detected between age and the smaller lateral angle but a significant (*P* < 0.0001) positive correlation between age and the MCA bifurcation angle in patients without MCA bifurcation aneurysms or in the total patients put together. No significant (*P* > 0.05) correlation was detected between age and the larger lateral angle.

No significant (*P* > 0.05) correlation was detected between age and the vascular angles at the MCA bifurcation in patients with MCA bifurcation aneurysms (Fig. 6). A significant (*P* < 0.0001) negative correlation was detected between age and the smaller lateral angle but a significant (*P* < 0.0001) positive correlation between age and the MCA bifurcation angle in patients without MCA bifurcation aneurysms or in the total patients put together (Fig. 6). No significant (*P* > 0.05) correlation was detected between age and the larger lateral angle in these patients.

**Figure 7:**
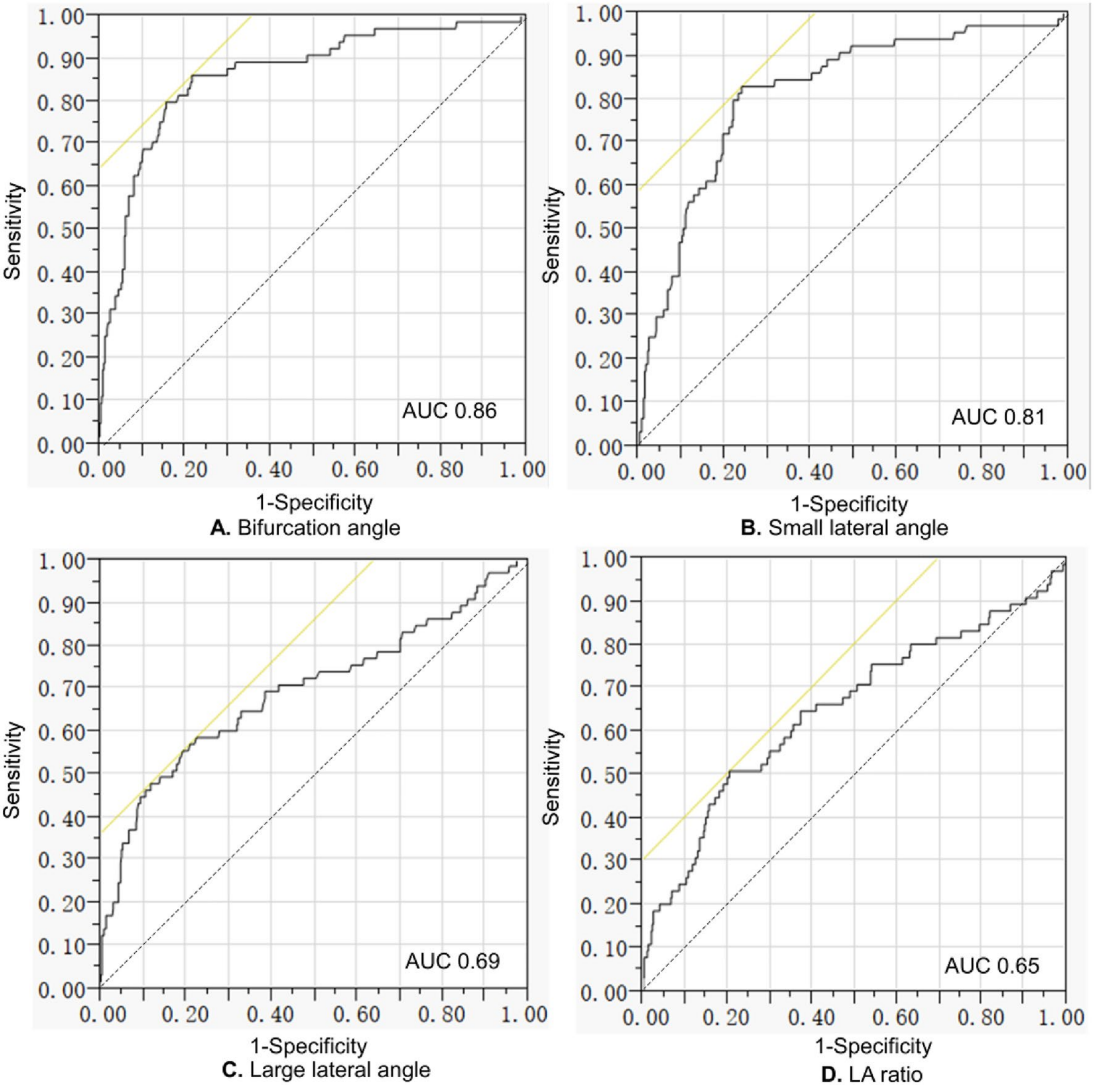
Receiver operating characteristic (ROC) curve analysis of the middle cerebral artery (MCA) bifurcation angle, small and large lateral angle, LA (larger lateral angle/smaller lateral angle) ratio, and aneurysm presence at the MCA bifurcation. (**A**) ROC curve analysis of the MCA bifurcation angle and aneurysm presence showed that the threshold value of the MCA bifurcation angle was 124.1° with an area under the ROC curve (AUC) of 0.86. (**B**) The threshold of the small lateral angle for predicting bifurcation aneurysm presence was 96.2° with an AUC of 0.81. (**C**) The threshold of the large lateral angle was 126.6° with an AUC of 0.69. (**D**) The threshold of the LA ratio was 1.56 with an AUC of 0.65.

In univariate logistic regression analysis (of smaller and larger lateral angle, bifurcation angle, diameter of the M1 and smaller and larger M2 artery, LA and DA ratio), the smaller and larger lateral angles, MCA bifurcation angle, and LA ratio were significant (*P* < 0.01) risk factors for presence of MCA bifurcation aneurysms. Significant parameters with the *P* value < 0.05 in the univariate analysis were entered in the multivariate logistic regression analysis after parameter adjustment, MCA bifurcation angle was the only significant (*P* = 0.0001, odds ratio 2.7, 95% confidence interval 1.6–3.8) independent risk factor for MCA bifurcation aneurysm presence. ROC curve analysis of significant continuous measurement data (MCA bifurcation angle, small and large lateral angle, and LA ratio) in predicting the presence of MCA bifurcation aneurysms showed that the MCA bifurcation angle threshold for aneurysm presence was 124.1° (Table 2 and Fig. 7), with an area under the ROC curve (AUC) of 0.86, a sensitivity 0.86, a specificity 0.78, a positive prediction value (PPV) 0.38, and a negative prediction value (NPV) 0.97. The threshold and AUC were 96.2° and 0.81 for small lateral angle, 126.6° and 0.69 for large lateral angle, and 1.56 and 0.65 for LA ratio, respectively (Table 2 and Fig. 7).

**Table 2:** Receiver operating characteristics (ROC) curve analysis for bifurcation aneurysm presence.

Variables	Threshold	AUC	Sensitivity	Specificity	PPV	NPV
Bifurcation angle	124.1°	0.86	0.86	0.78	0.38	0.97
Small lateral angle	96.2°	0.81	0.83	0.76	0.35	0.97
Large lateral angle	126.6°	0.69	0.55	0.81	0.31	0.92
LA ratio	1.56	0.65	0.51	0.79	0.28	0.91

*ROC* Receiver operating characteristics, *AUC* Area under the ROC curve, *PPV* Positive predictive value, *NPV* Negative predictive value, *LA* Larger lateral angle/smaller lateral angle.

## Discussion

In this study investigating the relationship of the MCA bifurcation aneurysms with patients’ age and sex, vascular angles at the bifurcation, and diameters of the M1 and two M2 arteries, it was found that significantly more MCA bifurcation aneurysms are present in older patients, females, and patients with a wider MCA bifurcation angle, and deviate towards the smaller lateral angle and the thinner M2 segment. MCA bifurcation angle is the only independent risk factor for presence of MCA bifurcation aneurysms with the threshold of 124.1°.

Our study found that women at the age of 40–70 years were more likely to suffer from the MCA bifurcation aneurysms. Other studies also found that women aged 40–70 years were more prone to presence of cerebral aneurysms at the basilar arterial bifurcation^7^ while women of 50–70 years of age were more vulnerable to aneurysm presence at the anterior cerebral arterial bifurcation (the anterior communicating artery complex)^6^. At this age range, the secretion of estrogen has been significantly decreased in women approaching the menopause or who have passed the menopause period. The estrogen can protect the arterial intima from damage by increased hemodynamic stresses, oxidative stress and hazardous nitric oxide synthase activity, and greatly dropped secretion of the estrogen will lose these protective effects on the arterial intima, leading to intimal injury, endothelial dysfunction, and subsequent aneurysm initiation and development^16–18^. Lack of the estrogen hormone is responsible for increased risks of aneurysm initiation and rupture in women.

Human extracellular matrix has an important component of elastin which provides resilience and elasticity to many tissues and organs, including the arteries and veins^19^. The elastin may provide great support for the integrity of the arterial wall, and degradation and deficiency of this material will cause arterial injury and aneurysm presence. Nonetheless, elastin has a half-life of over 70 years, and destruction of elastin will contribute to the initiation, development and deterioration of various pathological conditions like atherosclerosis, obstructive pulmonary diseases and cerebral aneurysms^17,19–21^. During the 40–70 year period, the elastin has been degraded in a large amount, leading to decreased elasticity and resilience of arteries and possible formation cerebral aneurysms.

Patients with aneurysms at the anterior cerebral artery bifurcation and basilar artery bifurcation have been revealed to have a wider bifurcation angle than those who do not have the bifurcation aneurysms^6,7,11^. Our study had also found a wider bifurcation angle in patients with the MCA bifurcation aneurysms than those without aneurysms (149.2° ± 32.6° vs. 107.2° ± 26.3°). The threshold of the MCA bifurcation angle was detected to be 124.1° with an AUC of 0.86. The intracranial vascular bifurcations possess a narrow dense collagen band which can provide stiffness and strength to protect the bifurcation apex wall from injury caused by abnormally-increased hemodynamic stresses^22,23^. In patients with wider bifurcation angles, the zone of increased hemodynamic stresses created by direct impingement of blood flow on the bifurcation apex may surpass the region protected by the narrow collagen band at the bifurcation, thus creating a larger area of flow impingement with greater insult of hemodynamic stresses and possibly leading to intimal damage and aneurysm initiation and formation. In addition, wider arterial bifurcations will affect the direction of blood flow and produce large strong vortices to damage the bifurcation wall, resulting in arterial wall bulge and aneurysm formation. In patients with narrowed bifurcation angles, the laminar flow may not be disturbed, and the possibility of aneurysm formation is decreased^6,7,13–15,24^.

Wider arterial bifurcation and abnormally-enhanced hemodynamic stresses on the bifurcation apex may induce an aneurysm based on the above discussion. Clinically, if the vascular bifurcation angle can be narrowed, the hemodynamic stresses will be decreased. So is the possibility of aneurysm formation at this site. Intracranial stents have been used in stent-assisted coiling of wide-necked cerebral aneurysms at the bifurcation^25–28^. With the stent being deployed at the bifurcation, the arterial bifurcation angle has been significantly decreased, and the hemodynamic stresses have also been reduced significantly, thus being able to protect the bifurcation apex from being damaged by abnormally-increased hemodynamic stresses^8,14^. Currently, intracranial stents have been used in the treatment of cerebral aneurysms at the bifurcations of the anterior cerebral artery, MCA, ICA, and basilar artery^8,14,25–29^, and use of intracranial stents at these cites is able to eliminate the hemodynamic pathogenic factors for aneurysm initiation and development.

Our study indicated that smaller parent artery diameter and M2 diameters were associated with MCA bifurcation aneurysms. Smaller arterial diameters have also been demonstrated to relate to aneurysms at the bifurcation of the anterior cerebral artery and basilar artery^6,7,11,30^. Alterations in the arterial diameter, bifurcation angle and symmetry may increase the strength of hemodynamic stresses to injure the arterial wall at the bifurcation apex^8,11,14,15,30^. This is probably the reason why the hemodynamic stresses are significantly minimized when the arterial diameter and bifurcation angle follow the optimality principle of minimum work^10,31,32^.

In our study, the maximal aneurysm size was significantly positively correlated with the diameter of M2 arteries. With increase in the M2 diameter, the room for aneurysm growth may also be significantly raised. Nonetheless, age was not in a significantly correlation with the arterial diameter. Our study did not detect a significant positive correlation in age with the MCA bifurcation angle in patients with the MCA bifurcation aneurysm, which is quite different from other studies^6,7,33^. In these studies^6,7,33^, age was significantly positively correlated with the bifurcation angle in patients with aneurysms at the bifurcation of basilar artery, MCA and anterior cerebral artery. Nonetheless, age was significantly positively correlated with the MCA bifurcation angle in 517 patients without any cerebral aneurysms or in all the 638 patients with and without aneurysms. This may indicate a lowered statistical power in fewer patients with MCA bifurcation aneurysms. Further studies with a large number of MCA bifurcation aneurysm patients will have to be involved to confirm this finding.

Our study had found that most (85%) of the MCA bifurcation aneurysms deviated towards the smaller lateral angle or the smaller M2 branch, which is in line with the findings of aneurysms at the bifurcations of the basilar artery and anterior cerebral artery^6,7^. Smaller lateral angle and thinner M2 branch at the MCA bifurcation may not follow the optimality principle of minimal work, and stronger hemodynamic stresses may be induced by the smaller magnitude of angles and branches at the bifurcation apex to initiate an aneurysm^10,31,32^.

Taken together, the MCA bifurcation aneurysms may be induced and developed by the following factors. As ageing, the MCA bifurcation angle significantly increased, thus contributing to the production of a bigger zone of significantly greater hemodynamic stresses beyond the protective area by the narrow collagen band at the bifurcation apex. Degradation of most of the elastin of the bifurcation apex wall at the age 40–70 years will lead to elasticity loss, bulge out of the arterial wall, and ultimately formation of an aneurysm at the bifurcation.

The findings of this study have some clinical significance. Older patients, especially females, at the age of 40–70 years with a wider MCA bifurcation greater than 124.1° should be highly suspected of development of a MCA bifurcation aneurysm sometime in the future and, thus, should be arranged for periodical cerebral angiographic monitor. In endovascular treatment of the MCA bifurcation aneurysm, the MCA bifurcation angle should be made narrower if treated with stent-assisted coiling, with the stent being deployed from the M1 segment to the thinner M2 segment or to the M2 segment which forms a smaller lateral angle with the M1 segment. In this way, the bifurcation angle will be significantly narrowed, and the hemodynamic stresses inducing formation of the MCA bifurcation aneurysm will be significantly altered, consequently protecting the MCA bifurcation apex wall from being damaged by the hemodynamic stresses and ensuring an endured treatment effect. For neurosurgeons treating MCA bifurcation aneurysms, measures may also be taken to narrow the MCA bifurcation angle so as to decrease the hemodynamic stresses from damaging the bifurcation apex, which may also ensure a long-time treatment effect. Some limitations existed in this study, including the retrospective and one-center study design, Chinese patients enrolled, a small cohort of patients with the MCA bifurcation aneurysms, no randomization or blindness, which may all affect the outcome of the study. Further prospective, randomized, controlled, multi-center studies involving multiple races and ethnicities will have to be conducted for better outcomes.

In conclusion, significantly more MCA bifurcation aneurysms are present in older patients, females, and patients with a wider MCA bifurcation angle, and deviate towards the smaller lateral angle and the thinner M2 segment. MCA bifurcation angle is the only independent risk factor for presence of MCA bifurcation aneurysms with the threshold of 124.1°.

## Data Availability

The datasets used and/or analysed during the current study available from the corresponding author on reasonable request.

## References

[1] Zhu W, Liu P, Tian Y, Gu Y, Xu B, Chen L (2013). Complex middle cerebral artery aneurysms: A new classification based on the angioarchitecture and surgical strategies. Acta Neurochir. (Wien)..

[2] Sadatomo T, Yuki K, Migita K, Imada Y, Kuwabara M, Kurisu K (2013). Differences between middle cerebral artery bifurcations with normal anatomy and those with aneurysms. Neurosurg. Rev..

[3] Inagawa T, Hirano A (1990). Autopsy study of unruptured incidental intracranial aneurysms. Surg. Neurol..

[4] Investigators UJ, Morita A, Kirino T, Hashi K, Aoki N, Fukuhara S (2012). The natural course of unruptured cerebral aneurysms in a Japanese cohort. N. Engl. J. Med..

[5] Link TW, Boddu SR, Hammad HT, Knopman J, Lin N, Gobin P (2018). Endovascular treatment of middle cerebral artery aneurysms: A single center experience with a focus on thromboembolic complications. Interv. Neuroradiol..

[6] Zhang XJ, Gao BL, Hao WL, Wu SS, Zhang DH (2018). Presence of anterior communicating artery aneurysm is associated with age, bifurcation angle, and vessel diameter. Stroke..

[7] Zhang XJ, Gao BL, Li TX, Hao WL, Wu SS, Zhang DH (2018). Association of basilar bifurcation aneurysms with age, sex, and bifurcation geometry. Stroke..

[8] Gao B, Baharoglu MI, Malek AM (2013). Angular remodeling in single stent-assisted coiling displaces and attenuates the flow impingement zone at the neck of intracranial bifurcation aneurysms. Neurosurgery..

[9] Ingebrigtsen T, Morgan MK, Faulder K, Ingebrigtsen L, Sparr T, Schirmer H (2004). Bifurcation geometry and the presence of cerebral artery aneurysms. J. Neurosurg..

[10] Zhang XJ, Li CH, Hao WL, Zhang DH, Gao BL (2019). The optimality principle decreases hemodynamic stresses for aneurysm initiation at anterior cerebral artery bifurcations. World Neurosurg..

[11] Zhang XJ, Li CH, Hao WL, Zhang DH, Ren CF, Gao BL (2018). Enlarged anterior cerebral artery bifurcation angles may induce abnormally enhanced hemodynamic stresses to initiate aneurysms. World Neurosurg..

[12] Chalouhi N, Hoh BL, Hasan D (2013). Review of cerebral aneurysm formation, growth, and rupture. Stroke..

[13] Baharoglu MI, Lauric A, Safain MG, Hippelheuser J, Wu C, Malek AM (2014). Widening and high inclination of the middle cerebral artery bifurcation are associated with presence of aneurysms. Stroke..

[14] Gao B, Baharoglu MI, Cohen AD, Malek AM (2013). Y-stent coiling of basilar bifurcation aneurysms induces a dynamic angular vascular remodeling with alteration of the apical wall shear stress pattern. Neurosurgery..

[15] Gao BL, Hao WL, Ren CF, Li CH, Wang JW, Liu JF (2022). Greater hemodynamic stresses initiated the anterior communicating artery aneurysm on the vascular bifurcation apex. J. Clin. Neurosci..

[16] Hoh BL, Rojas K, Lin L, Fazal HZ, Hourani S, Nowicki KW (2018). Estrogen deficiency promotes cerebral aneurysm rupture by upregulation of th17 cells and interleukin-17a which downregulates e-cadherin. J. Am. Heart Assoc..

[17] Maekawa H, Serrone JC, Tjahjadi M, Hernesniemi J (2016). The role of estrogen on the pathology of cerebral aneurysms. Expert Rev. Neurother..

[18] Sheinberg DL, McCarthy DJ, Elwardany O, Bryant JP, Luther E, Chen SH (2019). Endothelial dysfunction in cerebral aneurysms. Neurosurg. Focus.

[19] Heinz A (2020). Elastases and elastokines: Elastin degradation and its significance in health and disease. Crit. Rev. Biochem. Mol. Biol..

[20] Urry DW, Pattanaik A, Xu J, Woods TC, McPherson DT, Parker TM (1998). Elastic protein-based polymers in soft tissue augmentation and generation. J. Biomater. Sci. Polym. Ed..

[21] Zhu YQ, Li MH, Yan L, Tan HQ, Cheng YS (2014). Arterial wall degeneration plus hemodynamic insult cause arterial wall remodeling and nascent aneurysm formation at specific sites in dogs. J. Neuropathol. Exp. Neurol..

[22] Rowe AJ, Finlay HM, Canham PB (2003). Collagen biomechanics in cerebral arteries and bifurcations assessed by polarizing microscopy. J. Vasc. Res..

[23] Tutuncu F, Schimansky S, Baharoglu MI, Gao B, Calnan D, Hippelheuser J (2014). Widening of the basilar bifurcation angle: Association with presence of intracranial aneurysm, age, and female sex. J. Neurosurg..

[24] Hademenos GJ, Massoud TF (1997). Biophysical mechanisms of stroke. Stroke..

[25] Kashkoush A, El-Abtah ME, Srivatsa S, Desai A, Davison M, Achey R (2022). Comparative effectiveness of stent-assisted coiling and woven endobridge embolization for the treatment of unruptured wide-neck bifurcation intracranial aneurysms. J. Neurosurg..

[26] Suleyman K, Korkmazer B, Kocer N, Islak C, Kizilkilic O (2023). Evaluation of short- and long-term results of y-stent-assisted coiling with leo stents in endovascular treatment of wide-necked intracranial bifurcation aneurysms. Neuroradiology..

[27] Wan H, Lu G, Huang L, Ge L, Jiang Y, Zhang X (2023). Comparison of solitaire and neuroform stenting for coiling of intracranial bifurcation aneurysms. Interv. Neuroradiol..

[28] Liu C, Guo K, Wu X, Wu L, Cai Y, Hu X (2023). Utility of low-profile visualized intraluminal support (lvis) stent for treatment of acutely ruptured bifurcation aneurysms: A single-center study. Front. Neurol..

[29] Zhou Y, Yang PF, Hong B, Zhao WY, Huang QH, Li Q (2013). Stent placement for the treatment of complex internal carotid bifurcation aneurysms: A review of 16 cases. Turk. Neurosurg..

[30] Zhang XJ, Hao WL, Zhang DH, Gao BL (2019). Asymmetrical than symmetrical cerebral arterial bifurcations are more vulnerable to aneurysm presence. Sci. Rep..

[31] Alnaes MS, Isaksen J, Mardal KA, Romner B, Morgan MK, Ingebrigtsen T (2007). Computation of hemodynamics in the circle of willis. Stroke..

[32] Rossitti S, Lofgren J (1993). Optimality principles and flow orderliness at the branching points of cerebral arteries. Stroke..

[33] Zhang XJ, Hao WL, Zhang DH, Gao BL (2019). Asymmetrical middle cerebral artery bifurcations are more vulnerable to aneurysm formation. Sci. Rep..

